# Supplementary material for: Cognitive flexibility and sociality in Guinea baboons (Papio papio)
Source: PLoS One. 2024 Dec 19;19(12):e0308778. doi: 10.1371/journal.pone.0308778 (PMC11658514; doi:10.1371/journal.pone.0308778)
Supplement: S1 Table — (DOCX) [file pone.0308778.s001.docx]

S1 Table: Spearman correlation tests (and their confidence interval) on centrality and rank factors, among the 3 phases.

|  | **Correlations between EvC** | | **Correlation between Ranks** | |
| --- | --- | --- | --- | --- |
| Phases | A1 | A2 | A1 | A2 |
| A2 | rs = 0.70  CI = [0.25 ; 0.90] |  | rs = 0.85  CI = [0.54 ; 0.97] |  |
| A3 | rs = 0.87  CI = [0.60 ; 0.96] | rs = 0.74  CI = [0.34 ; 0.92] | rs = 0.9  CI = [0.64 ; 0.98] | rs = 0.97  CI = [0.87 ; 1] |
