# Supplementary material for: Cognitive flexibility and sociality in Guinea baboons (Papio papio)
Source: PLoS One. 2024 Dec 19;19(12):e0308778. doi: 10.1371/journal.pone.0308778 (PMC11658514; doi:10.1371/journal.pone.0308778)
Supplement: S2 Table — Note that the “OLD” age class was removed from the dataset in this analysis. (DOCX) [file pone.0308778.s002.docx]

S2 Table: Results of the linear mixed effect model on the average of perseverative errors on the first 50 trials after a rule change. Note that the “OLD” age class was removed from the dataset in this analysis.

**Formula: Mean Perseveration ~ Rank + EvC + Age class + (scale (Rule Sessions) | Name)**

| Random effects: | |  |  |  |  |  |
| --- | --- | --- | --- | --- | --- | --- |
|  | Groups | Name | Variance | Std.Dev. | Corr |  |
|  | Name | (Intercept) | 16.29 | 4.036 |  |  |
|  | scale(Session) | 17.34 | 4.164 | 0.98 |  |  |
|  | Residual | 11.74 | 3.426 |  |  |  |
|  |  |  |  |  |  |  |
| Fixed effects: | |  |  |  |  |  |
|  | Estimate | Std.Error | df | t | value | Pr(>\|t\|) |
| **(Intercept)** | **12.054803** | **1.539539** | **4.178946** | **7.830** | **0.00120** | ****** |
| Rank | -0.004123 | 0.046702 | 2.960014 | -0.088 | 0.93529 |  |
| EvC | -14.642242 | 5.645146 | 3.844564 | -2.594 | 0.06288 | . |
| **AgeClassAdult** | **-4.748827** | **0.630748** | **2.856371** | **-7.529** | **0.00574** | ****** |
| **AgeClassMiddleAge** | **-3.143737** | **0.670306** | **2.749127** | **-4.690** | **0.02221** | ***** |

Number of observations: 4080, groups: Name, 13
