# Supplementary material for: Cognitive flexibility and sociality in Guinea baboons (Papio papio)
Source: PLoS One. 2024 Dec 19;19(12):e0308778. doi: 10.1371/journal.pone.0308778 (PMC11658514; doi:10.1371/journal.pone.0308778)
Supplement: S3 Table — Note that the “OLD” age class was removed from the dataset in this analysis. (DOCX) [file pone.0308778.s003.docx]

S3 Table: Results of the linear mixed effect model on the learning latency after a rule shift. Note that the “OLD” age class was removed from the dataset in this analysis.

**Formula: Learning latency ~ Rank + EvC + Age class + (scale (Rule Sessions) | Name)**

| Random effects: | |  |  |  |  |  |
| --- | --- | --- | --- | --- | --- | --- |
| Groups | Name | Variance | Std.D |  |  |  |
| Name | (Intercept) | 105.1 | 10.25 |  |  |  |
| scale(Session) | 159.2 | 12.62 |  |  |  |  |
| Residual | 183.6 | 13.55 |  |  |  |  |
|  |  |  |  |  |  |  |
| Fixed effects: | |  |  |  |  |  |
|  | Estimate | Std.Error | df | t | value | Pr(>\|t\|) |
| **(Intercept)** | **53.7777** | **3.7485** | **4.3197** | **14.347** | **8.29e-05** | ******* |
| Rank | -0.2167 | 0.1059 | 3.5207 | -2.047 | 0.11956 |  |
| **EvC** | **-57.7992** | **13.3860** | **4.0598** | **-4.318** | **0.01207** | ***** |
| **AgeClassAdult** | **-13.5411** | **1.3930** | **3.3775** | **-9.721** | **0.00139** | ****** |
| **AgeClassMiddleAge** | **-9.6414** | **1.5048** | **3.4787** | **-6.407** | **0.00485** | ****** |

Number of observations: 4080, groups: Name, 13
