# Supplementary material for: Cognitive flexibility and sociality in Guinea baboons (Papio papio)
Source: PLoS One. 2024 Dec 19;19(12):e0308778. doi: 10.1371/journal.pone.0308778 (PMC11658514; doi:10.1371/journal.pone.0308778)
Supplement: S4 Table — Note that the “OLD” age class was removed from the dataset in this analysis. (DOCX) [file pone.0308778.s004.docx]

S4 Table: Results of the linear mixed effect model on the average RT on the first 50 trials after a rule. Note that the “OLD” age class was removed from the dataset in this analysis.

**Formula: Mean RT ~ Rank + EvC + Age class + (scale (Rule Sessions) | Name)**

| Random effects: | |  |  |  |  |  |
| --- | --- | --- | --- | --- | --- | --- |
|  | Groups | Name | Variance | Std.Dev. | Corr |  |
|  | Name | (Intercept) | 38710 | 196.7 |  |  |
|  | scale(Session) | 19051 | 138.0 | 0.81 |  |  |
|  | Residual | 47156 | 217.2 |  |  |  |
|  |  |  |  |  |  |  |
| Fixed |  |  |  |  |  |  |
|  | Estimate | Std.Error | df | t | value | Pr(>\|t\|) |
| (Intercept) | 1847.661 | 151.367 | 9.656 | 12.206 | 3.47e-07 | *** |
| Rank | 1.438 | 6.173 | 5.967 | 0.233 | 0.82356 |  |
| EvC | -1795.785 | 566.498 | 10.473 | -3.170 | 0.00945 | ** |
| AgeClassAdult | -416.049 | 80.787 | 6.366 | -5.150 | 0.00177 | ** |
| AgeClassMiddleAge | -106.585 | 88.686 | 6.405 | -1.202 | 0.27197 |  |

Number of observations: 4080, groups: Name, 13
